# Electrocardiographic Risk Stratification in COVID-19 Patients

**DOI:** 10.3389/fcvm.2021.636073

**Published:** 2021-02-02

**Authors:** Ehud Chorin, Matthew Dai, Edward Kogan, Lalit Wadhwani, Eric Shulman, Charles Nadeau-Routhier, Robert Knotts, Roi Bar-Cohen, Chirag Barbhaiya, Anthony Aizer, Douglas Holmes, Scott Bernstein, Michael Spinelli, David Park, Larry Chinitz, Lior Jankelson

**Affiliations:** Leon H. Charney Division of Cardiology, Cardiac Electrophysiology, NYU Langone Health, New York University School of Medicine, New York City, NY, United States

**Keywords:** predictors, mortality, troponin, COVID−19, ECG

## Abstract

**Background:** The COVID-19 pandemic has resulted in worldwide morbidity at unprecedented scale. Troponin elevation is a frequent laboratory finding in hospitalized patients with the disease, and may reflect direct vascular injury or non-specific supply-demand imbalance. In this work, we assessed the correlation between different ranges of Troponin elevation, Electrocardiographic (ECG) abnormalities, and mortality.

**Methods:** We retrospectively studied 204 consecutive patients hospitalized at NYU Langone Health with COVID-19. Serial ECG tracings were evaluated in conjunction with laboratory data including Troponin. Mortality was analyzed in respect to the degree of Troponin elevation and the presence of ECG changes including ST elevation, ST depression or T wave inversion.

**Results:** Mortality increased in parallel with increase in Troponin elevation groups and reached 60% when Troponin was >1 ng/ml. In patients with mild Troponin rise (0.05–1.00 ng/ml) the presence of ECG abnormality and particularly T wave inversions resulted in significantly greater mortality.

**Conclusion:** ECG repolarization abnormalities may represent a marker of clinical severity in patients with mild elevation in Troponin values. This finding can be used to enhance risk stratification in patients hospitalized with COVID-19.

## Introduction

Coronavirus Disease (COVID19) pandemic, induced by severe acute respiratory syndrome coronavirus-2 (SARS-CoV-2), is reaching now historical magnitude as one of the deadliest outbreaks in modern history (1). As of December 30 2020, over 80 million individuals were reported to be infected by SARS-CoV-2, with more than 1.8 million deaths (2). Recent reports (3, 4) revealed that cardiac complications are common (≈20–25%) in COVID19 infection and are associated with increased mortality. However, in those reports, “cardiac complications” were defined according to clinical and laboratory parameters (troponin levels) without systematic electrocardiographic (ECG) evaluation. It is unknown if elevations in troponin levels are reflective of a primary myocardial infarction, supply-demand inequity, or non-ischemic direct myocardial injury. The ECG is an attractive diagnostic tool as it is widely available and can be rapidly performed without inducing significant exposure of caregivers to SARS-CoV-2. ECG has been demonstrated to aid with prognostication in population-based studies (5, 6) and thus offers a particularly appealing modality during the current pandemic. We thus sought to determine whether findings on the first presenting ECG provide prognostic information and provide insights on myocardial injury. We reviewed ECGs of consecutive patients with COVID19 infection requiring hospitalization. We examined our findings stratified by troponin levels and clinical condition.

## Methods

This is a retrospective study performed at NYU Langone Medical Center, New York, USA. We included 204 consecutive adult patients hospitalized at NYU Langone Medical Center with COVID19 disease. Medical records were reviewed to obtain baseline characteristics, laboratory data, and ECGs. Troponin I concentrations were assessed via the Abbott Architect method (Abbott, Abbott Park, Illinois) wherein the 99th percentile for a normal population is 0.05 ng/mL and the maximal Troponin level was recorded. Descriptive analyses were performed by troponin levels stratified into normal (0.00–0.05 ng/mL), mildly elevated (0.05–1 ng/mL), and significantly elevated (>1 ng/mL). The first, presenting ECGs were reviewed and interpreted by five senior cardiologists who were blinded to the clinical status of the patients. Data reviewed from each ECG included heart rate, rhythm categorized as normal sinus rhythm or atrial fibrillation/flutter (AF), atrioventricular block (AVB), right bundle branch block (RBBB), left bundle branch block (LBBB), a non-specific intraventricular conduction block (QRS duration >120 ms), the presence of ST segment or T-wave changes (localized ST elevation, localized T-wave inversion, or other non-specific repolarization abnormalities). The closing date of follow-up was April 15th 2020. Collected data on the closing date included arrhythmic events and mortality. The study was reviewed and approved by the NYU Institutional Review Board and Quality Improvement initiative in accordance with the ethical standards laid down in the 1964 Declaration of Helsinki and its later amendments, with a waiver of informed consent.

### Statistical Analysis

Statistical analysis was performed using IBM SPSS Statistics 26, and figures were constructed using GraphPad Prism 8. Continuous variables are expressed as mean ± standard deviation or median (25th−75th percentile), and categorical variables are expressed as count (percentages). Normality of data samples was assessed using Shapiro-Wilk test. Two sample hypothesis testing for continuous variables was performed using Student's *t*-test if samples had normal distributions and Mann-Whitney U test if samples did not have normal distributions. Hypothesis testing for categorical variables was performed using Fisher's exact test. Significance testing for Kaplan-Meier curves was performed using log-rank test. For predictors of mortality, univariate analysis was performed using Cox proportional hazards regression, and significant univariate predictors were included in the multivariate analysis.

## Results

**Figure 1:**
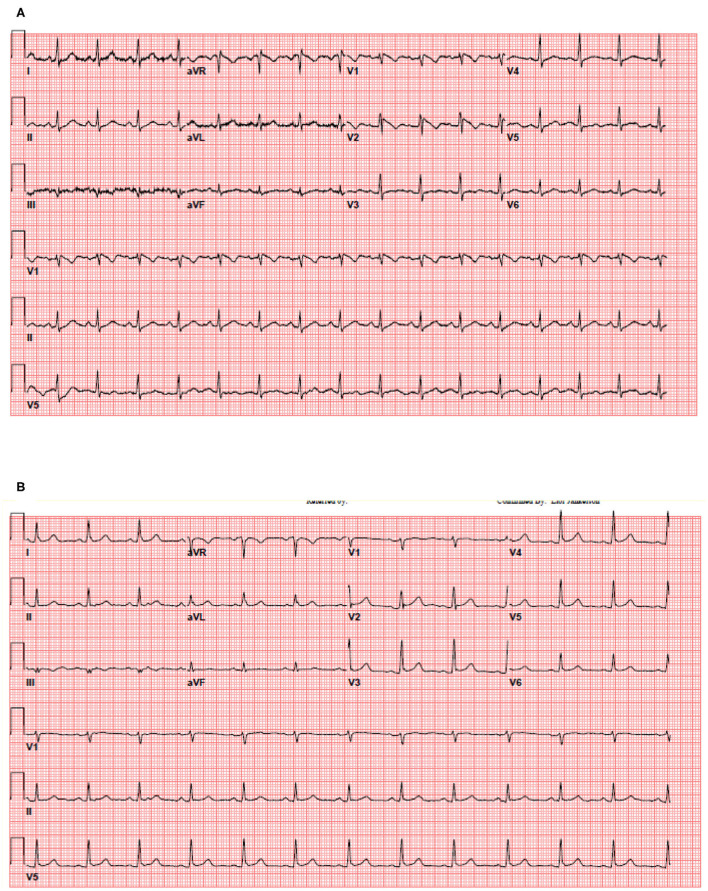
Thirty five year old female patient without significant medical history presented with a fever of 103.1 F. **(A)** The patient's initial 12-lead electrocardiogram in the emergency department. **(B)** The patient's repeat 12-lead electrocardiogram with resolution of fever.

**Figure 2:**
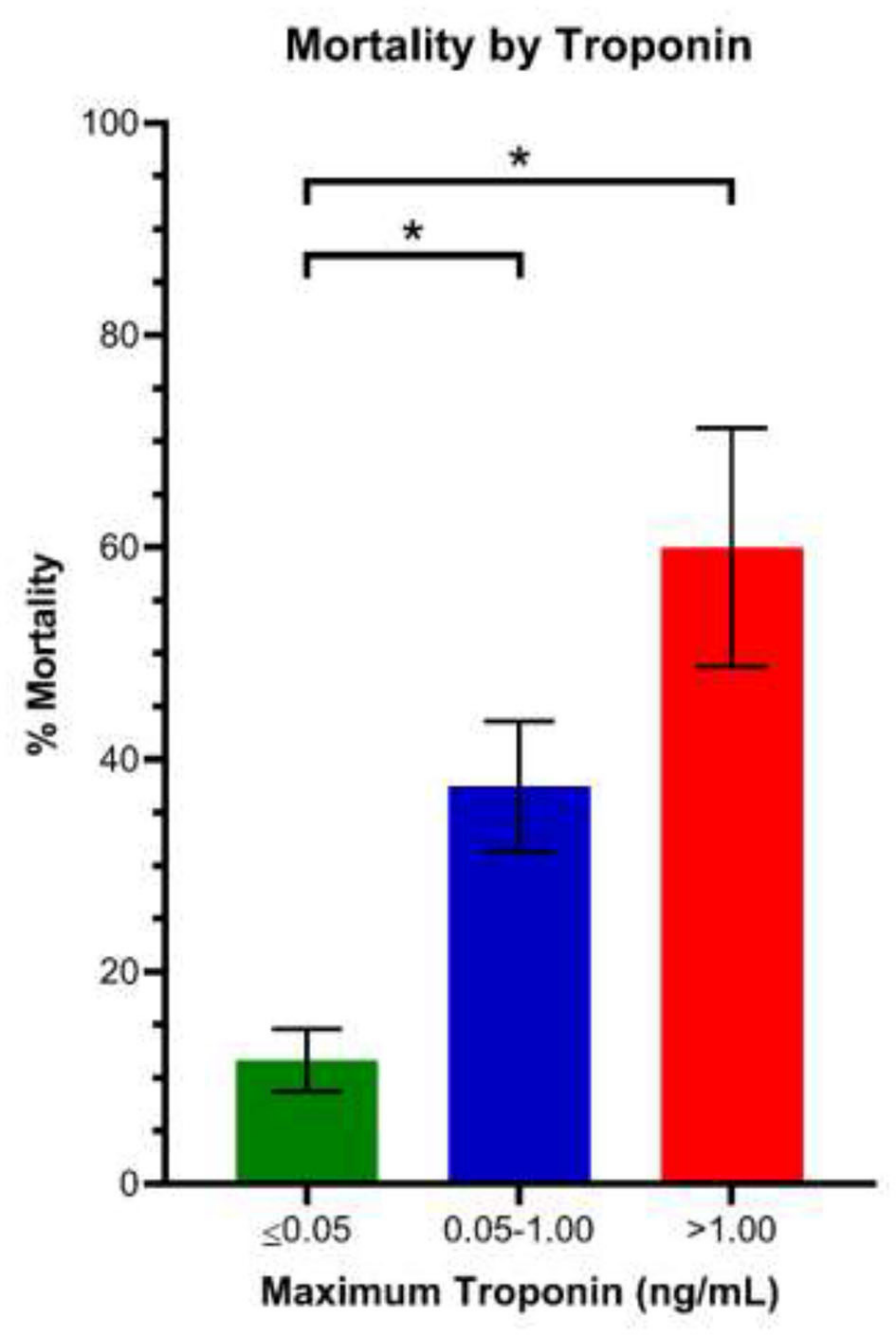
Mortality rate stratified by Troponin elevation groups. *P < 0.01.

**Figure 3:**
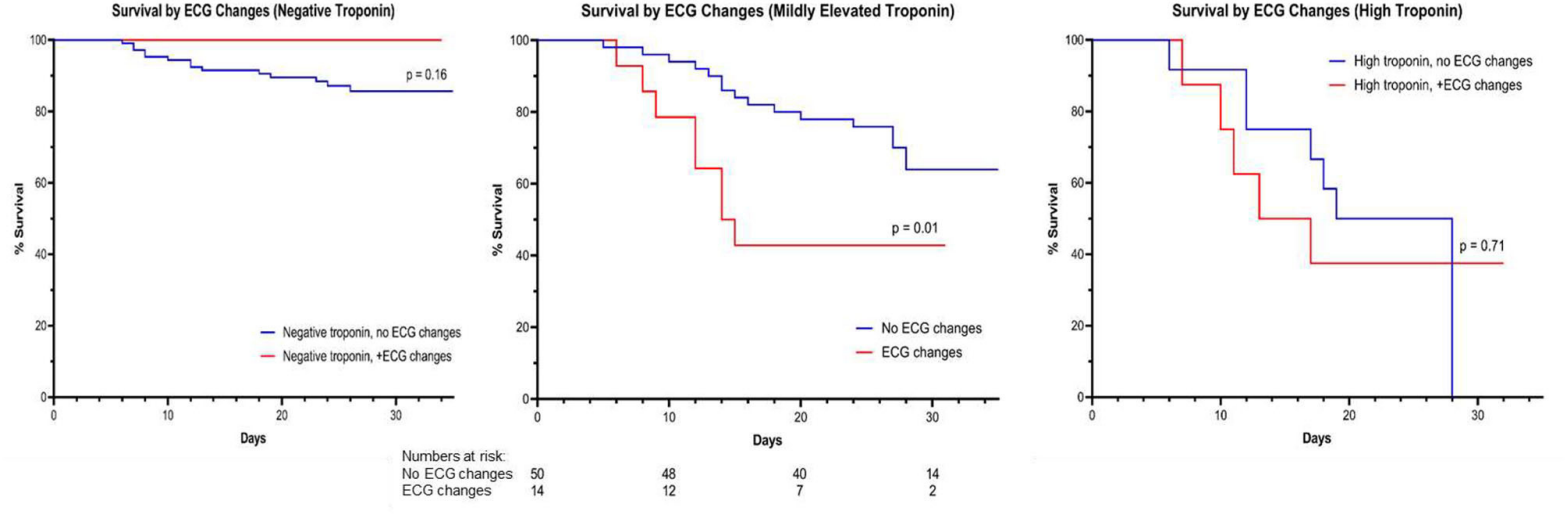
KM survival according to ECG changes stratified by Troponin elevation group.

We included 204 patients in our cohort with a mean follow up time of 24.2 ± 7.4 days. The clinical and epidemiological characteristics stratified by ECG abnormalities are presented in Table 1. The mean age was 64 ± 13 years and 76% were male. Comorbidities were common: 30% of patients had diabetes mellitus, 56% had hypertension, 12% had coronary artery disease, 3% had heart failure, and 6% had chronic obstructive pulmonary disease (COPD). Baseline electrocardiographic characteristics revealed mean HR of 89 ± 16 bpm and mean Bazett-corrected QT interval of 444 ± 26 ms. The vast majority were in normal sinus rhythm (95%), while 5% of patients had AF. Atrioventricular block was rare: 9 (4%) patients had a first degree AV block and no patients had second or third degree AV block. Abnormal intraventricular conduction was found in 11% (with RBBB in 8%, LBBB in 3%). Repolarization abnormalities (ST elevation, ST depression, or T wave inversion) were common (36 patients, 17.6%): one patient (0.5%) had localized ST elevation, 12 (5.9%) had ST depression, and 28 (13.7%) had localized T-wave inversion. Patients with repolarization abnormalities demonstrated higher troponin levels and a trend toward higher mortality (Table 1). One patient presented with a fever of 103.1 F which unmasked a previously unknown type I Brugada pattern (Figure 1). Fifty (25%) patients died of respiratory or multi-organ failure. In univariate and multivariate Cox regression analyses, clinical predictors of death included age and elevated Troponin (Table 2), but did not include gender, race or cardiovascular comorbidities (CAD, CHF, HTN). The mortality rate increased with incrementally higher troponin group: 14/120 [11.7%] for patients with negative troponin, 24/64 [37.5%] for patients with mildly elevated troponin, and 12/20 [60%], for patients with significantly elevated troponin (*p* < 0.01; Figure 2). The presence of an abnormal ECG finding resulted in significantly lower survival in the intermediate Troponin elevation group (0.05–1 ng/ml) but not in the low (<0.05 ng/ml) or high (> 1 ng/ml) Troponin elevation groups (Figure 3). In multivariate regression analysis, T wave inversion but not ST depression remained a significant predictor of mortality (HR 2.71, 95% CI 1.01–7.25, *p* = 0.04) in the intermediate Troponin group.

**Table 1:** Baseline characteristics of patients with COVID-19.

	**Overall** **(*n* = 204)**	**ECG changes** **(*n* = 36)**	**No ECG changes** **(*n* = 168)**	***p***
Age (years)	64 ± 13	67 ± 13	63 ± 13	0.14
Gender (% male)	156 (76%)	25 (69%)	131 (78%)	0.28
Race				0.08
African-American	10 (5%)	4 (11%)	6 (4%)	
Non-AA	191 (95%)	31 (89%)	160 (96%)	
Weight (kg)	86.6 ± 17.6	83.7 ± 16.4	87.3 ± 17.8	0.25
CAD	25 (12%)	7 (19%)	18 (11%)	0.16
HTN	114 (56%)	20 (56%)	94 (56%)	1
CKD	17 (8%)	6 (17%)	11 (7%)	0.09
DM	61 (30%)	18 (50%)	43 (26%)	<0.01
COPD	13 (6%)	4 (11%)	9 (5%)	0.25
CHF	7 (3%)	2 (6%)	5 (3%)	0.61
Initial creatinine (mg/dL)	1.3 ± 1.0	1.6 ± 1.4	1.2 ± 0.9	0.57
Abnormal LFTs	48 (25%)	10 (29%)	38 (24%)	0.51
Initial troponin (ng/mL)	0.02 (0.01 - 0.04)	0.02 (0.01 - 0.08)	0.02 (0.01 - 0.04)	0.17
Maximum troponin	0.04 (0.01 - 0.15)	0.12 (0.02 - 0.47)	0.03 (0.01 - 0.11)	0.01
Troponin group				<0.01
≤ 0.05	120 (59%)	14 (39%)	106 (63%)	
0.05–1.00	64 (31%)	14 (39%)	50 (30%)	
>1.00	20 (10%)	8 (22%)	12 (7%)	
Mortality	50 (23%)	13 (36%)	37 (22%)	0.09

**Table 2:** Predictors of mortality in patients with COVID-19.

**Variable**	**HR**	**95% CI**	***p-*value**
**UNIVARIATE REGRESSIONS**			
Age (years)	1.08	1.05–1.1	<0.01
Gender (% male)	1.09	0.56–2.13	0.80
**RACE**			
African-American	0.8	0.2–3.3	0.76
Weight (kg)	0.99	0.97–1.01	0.18
CAD	1.08	0.49–2.41	0.84
HTN	1.39	0.78–2.48	0.26
CKD	2.4	1.12–5.11	0.02
DM	1.77	1.01–3.1	0.05
COPD	1.46	0.53–4.07	0.47
CHF	2.09	0.65–6.73	0.22
Initial creatinine (mg/dL)	1.24	1.08–1.43	<0.01
Abnormal LFTs	0.84	0.42–1.7	0.64
Initial troponin (ng/mL)	0.99	0.84–1.16	0.91
Maximum troponin	1.01	1.0–1.02	0.02
Positive troponin (>0.05)	4.24	2.28–7.86	<0.01
**MULTIVARIATE REGRESSION**			
Age (years)	1.06	1.04–1.1	<0.01
CKD	1.53	0.65–3.6	0.33
DM	0.99	0.53–1.86	0.98
Positive troponin (>0.05)	3.22	1.71–6.05	<0.01

## Discussion

Multiple mechanisms have been shown to explain the frequent COVID-19 induced cardiovascular injury. These include direct injury to the myocardium induced by a cytokine storm resulting from a hyperinflammatory state, microvascular damage resulting from abnormal activation of the coagulation cascade including disseminated intravascular coagulation and thrombosis, supply-demand mismatch resulting from respiratory induced tissue hypoxia in conjunction with increase in metabolic demand of infection and inflammation, and myocardial injury by direct entry of SARS-CoV-2 into cardiomyocytes expressing the ACE2 receptor (7–9). In this retrospective cohort study we further assess the interaction of ECG abnormalities and Troponin elevation. We demonstrate that (1) myocardial injury defined by elevated Troponin is indeed prevalent in patients hospitalized with COVID-19 but is more often mild, associated with low-level elevation in troponin concentration. (2) more significant myocardial injury, as evident by increased Troponin level may be associated with higher risk of mortality. (3) In the group of patients with mild Troponin elevation (0.05–1 ng/ml), ECG abnormalities, and particularly T wave inversions are associated with significantly increased mortality. Consistent with our findings, a recent study had demonstrated that T wave inversion is highly frequent finding in patients with COVID-19, conferring increased risk for mortality and particularly when accompanied by Troponin elevation (10).

Though troponin elevation above the 99th percentile of the upper reference limit is considered the central marker of “myocardial injury,” mild elevation between 0.05 and 1 is often non-specific and associated with non-vascular etiologies such as strain, myocyte necrosis and increased cell membrane permeability (11–13). Indeed, mild Troponin elevation was a frequent finding in our cohort, present in 31% of patients with COVID-19. In this regard, our data suggests that assessment for the presence of ECG abnormalities can be used to enhance inpatient risk stratification in those patients with mild Troponin elevation, with potentially intensification of monitoring and therapy. Finally, as persistent fever is a frequent clinical feature of COVID-19, as well as potential side effect of the novel vaccines, caregivers should be familiar with the phenomena of fever induced Brugada pattern and not mistake it for ST elevation myocardial infarction. For patients who present with transient, fever induced Brugada pattern, elective challenge with sodium channel blocking agent (Procainamide, Flecainide, Ajmalin) after resolution of the acute illness can establish the diagnosis of Brugada.

## Limitations

Our study has several limitations. This is an observational, retrospective study. Because of its retrospective nature, the study is subject to selection bias, and its results imply association, not cause and effect. Relatively short follow-up time after was available. The study was not aimed at providing mechanistic insight for the cause of repolarization changes and Troponin elevation. We did not asses structural information from echo due to limited number of tests performed. However, this study was directed at assessing the presenting ECG as a readily available tool for risk stratification in combination with Troponin, a simple blood test.

## Data Availability Statement

The data analyzed in this study is subject to the following licenses/restrictions: Data sharing will be considered pending request. Requests to access these datasets should be directed to lior.jankelson@nyumc.org.

## Ethics Statement

The studies involving human participants were reviewed and approved by Quality Control Act With Accordance to the NYU Langone Health IRB. Written informed consent for participation was not required for this study in accordance with the national legislation and the institutional requirements.

## Author Contributions

LJ and EC: concept, data collection, and manuscript. MD and EK: statistics. LW, ES, CN-R, RK, and RB-C: data collection. CB, AA, DH, SB, MS, DP, and LC: manuscript writing and review. All authors contributed to the article and approved the submitted version.

## Conflict of Interest

The authors declare that the research was conducted in the absence of any commercial or financial relationships that could be construed as a potential conflict of interest.

## Abbreviations

ECG: Electrocardiography
COPD: Chronic obstructive pulmonary disease
CAD: Coronary artery disease
CHF: Congestive heart failure
CKD: Chronic kidney disease
HTN: Hypertension
DM: Diabetes mellitus
LFTs: Liver function tests.

## References

[1] Huang C Wang Y Li X Ren L Zhao J Hu Y Zhang L. Clinical features of patients infected with 2019 novel coronavirus in Wuhan, China. Lancet. (2020) 395:497–506. 10.1016/S0140-6736(20)30183-5 31986264 PMC7159299

[2] WHO Coronavirus Disease (COVID-19) Pandemic. Geneva: World Health Organization (2020). Available online at: https://www.who.int/emergencies/diseases/novel-coronavirus-2019

[3] Zhou F Yu T Du R Fan G Liu Y Liu Z Clinical course and risk factors for mortality of adult inpatients with COVID-19 in Wuhan, China: a retrospective cohort study. Lancet. (2020) 395:1054–62. 10.1016/S0140-6736(20)30566-3 32171076 PMC7270627

[4] Guo T Fan Y Chen M Wu X Zhang L He T. cardiovascular implications of fatal outcomes of patients with coronavirus disease 2019 (COVID-19). JAMA Cardiol. (2020) 5:811–18. 10.1001/jamacardio.2020.1017 32219356 PMC7101506

[5] Rautaharju PM Kooperberg C Larson JC LaCroix A Electrocardiographic predictors of incident congestive heart failure and all-cause mortality in post-menopausal women: the Women's Health Initiative. Circulation. (2006) 113:481–9. 10.1161/CIRCULATIONAHA.105.537415 16449727

[6] Daviglus ML Liao Y Greenland P Dyer AR Liu K Xie X. Association of nonspecific minor ST-T abnormalities with cardiovascular mortality: the Chicago Western electric study. JAMA. (1999) 282:530–6. 10.1001/jama.281.6.530 10022109

[7] Clerkin KJ Fried JA Raikhelkar J Sayer G Griffin JM Masoumi A. Coronavirus disease 2019 (COVID-19) and cardiovascular disease. Circulation. (2020) 140:1648–55. 10.1161/CIRCULATIONAHA.120.046941 32200663

[8] Patel AB Verma A COVID-19 and angiotensin-converting enzyme inhibitors and angiotensin receptor blockers: what is the evidence? JAMA. (2020) 323:1769–1770. 10.1001/jama.2020.4812 32208485

[9] Madjid M Safavi-Naeini P Solomon SD Vardeny O. Potential effects of coronaviruses on the cardiovascular system: a review. JAMA Cardiology. (2020) 5:831–40. 10.1001/jamacardio.2020.1286 32219363

[10] Korff S Katus HA Giannitsis E. Differential diagnosis of elevated troponins. Heart. (2006) 92:987–93. 10.1136/hrt.2005.071282 16775113 PMC1860726

[11] Romero J Alviz I Parides M Diaz JC Briceno D Gabr M. T-wave inversion as a manifestation of COVID-19 infection: a case series. J Interv Card Electrophysiol. (2020) 59:485–93. 10.1007/s10840-020-00896-7 33128658 PMC7602831

[12] Januzzi JL Jr McCarthy CP. Trivializing an elevated troponin: adding insult to injury? J Am Coll Cardiol. (2019) 73:10–2. 10.1016/j.jacc.2018.10.042 30621938

[13] Park KC Gaze DC Collinson PO Marber MS. Cardiac troponins: from myocardial infarction to chronic disease. Cardiovasc Res. (2017) 113:1708–18.e13. 10.1093/cvr/cvx183 29016754 PMC5852618

